# Polyhydroxyalkanoates from a Mixed Microbial Culture: Extraction Optimization and Polymer Characterization

**DOI:** 10.3390/polym14112155

**Published:** 2022-05-25

**Authors:** Ana Marta Rodrigues, Rita Dias Guardão Franca, Madalena Dionísio, Chantal Sevrin, Christian Grandfils, Maria A. M. Reis, Nídia Dana Lourenço

**Affiliations:** 1 Associate Laboratory i4HB–Institute for Health and Bioeconomy, NOVA School of Science and Technology, NOVA University Lisbon, 2829-516 Caparica, Portugal; amsi.rodrigues@campus.fct.unl.pt (A.M.R.); r.franca@campus.fct.unl.pt (R.D.G.F.); amr@fct.unl.pt (M.A.M.R.); 2 UCIBIO—Applied Molecular Biosciences Unit, Department of Chemistry, NOVA School of Science and Technology, NOVA University Lisbon, 2829-516 Caparica, Portugal; 3 LAQV-REQUIMTE, Department of Chemistry, NOVA School of Science and Technology, NOVA University Lisbon, 2829-516 Caparica, Portugal; madalena.dionisio@fct.unl.pt; 4 CEIB-Interfaculty Research Centre of Biomaterials, University of Liege, B-4000 Liège, Belgium; csevrin@uliege.be (C.S.); c.grandfils@uliege.be (C.G.)

**Keywords:** PHA extraction, chemical digestion, design of experiments, polymer properties

## Abstract

Polyhydroxyalkanoates (PHA) are biopolymers with potential to replace conventional oil-based plastics. However, PHA high production costs limit their scope of commercial applications. Downstream processing is currently the major cost factor for PHA production but one of the least investigated aspects of the PHA production chain. In this study, the extraction of poly(3-hydroxybutyrate-co-3-hydroxyvalerate) produced at pilot scale by a mixed microbial culture was performed using sodium hydroxide (NaOH) or sodium hypochlorite (NaClO) as digestion agents of non-PHA cellular mass. Optimal conditions for digestion with NaOH (0.3 M, 4.8 h) and NaClO (9.0%, 3.4 h) resulted in polymers with a PHA purity and recovery of ca. 100%, in the case of the former and ca. 99% and 90%, respectively, in the case of the latter. These methods presented higher PHA recoveries than extraction by soxhlet with chloroform, the benchmark protocol for PHA extraction. The polymers extracted by the three methods presented similar PHA purities, molecular weights and polydispersity indices. Using the optimized conditions for NaOH and NaClO digestions, this study analyzed the effect of the initial intracellular PHA content (40–70%), biomass concentration (20–100 g/L) and biomass pre-treatment (fresh vs. dried vs. lyophilized) on the performance of PHA extraction by these two methods.

## 1. Introduction

Polyhydroxyalkanoates (PHA) are biodegradable and biobased polymers produced by bacterial fermentation. PHA accumulate intracellularly as water insoluble inclusion bodies that function as carbon and energy storage [1,2,3], The replacement of conventional oil-based plastics by PHA in various segments has been thoroughly discussed due to their similar thermo-physical properties, with PHA offering environmentally friendly advantages, such as biocompatibility, biodegradability and compostability [1,4]. PHA production costs, however, are currently higher than those for oil-derived plastics (EUR 1.18–6.12/kg vs. <EUR 1/kg), which limits their potential to niche-market high value applications [1]. Continuous efforts have been made to reduce PHA production costs by applying mixed microbial cultures (MMC) and waste streams as carbon source for PHA production, as an alternative to pure cultures and the required sterile conditions and expensive substrates. Hence, PHA downstream processing is now the most economically impacting factor in the PHA production chain [1,4].

The best established and most commonly used methods for PHA recovery employ halogenated solvents, such as chloroform. Although these methods often generate superior recovery yields and product purity values, they are environmentally harmful, make use of large volumes of noxious solvents and require excessive energy input, making the recovery process unsustainable and economically unfeasible [4,5]. Thus, research has been leaning toward the development of more environmentally friendly approaches for the extraction and purification of PHA [4].

The use of alkaline compounds, such as sodium hydroxide (NaOH), can be a valid, cost-effective and green alternative to chlorinated compounds for the extraction of PHA [2]. Furthermore, alkaline treatment for PHA recovery has been considered more economically feasible when compared to an organic solvent-based process [6]. Hydroxides cause saponification of the lipids present in the cell wall of the microorganism, leading to increased membrane permeability and release of proteins and non-PHA cell material [7]. 

Mohammadi et al. (2012) [8] used NaOH to recover PHA (poly(3-hydroxybutyrate-co-3-hydroxyhexanoate), PHBHHx) from lyophilized recombinant *Cupriavidus necator* (PHA content of ca. 38.3%), having tested different NaOH concentrations, digestion times and reaction temperatures. The authors obtained over 96% of PHA recovery and purity by treating lyophilized cells with 0.05 M NaOH at 4 °C for 3 h. The fact that this efficient, simple, non-toxic and environmentally friendly treatment resulted in negligible degradation of the polymer molecular weight (Mw) supports the utilization of alkaline-based methods as an alternative to organic solvents for PHA recovery. Jiang et al. (2015) [3] assessed the feasibility of using NaOH for the extraction and purification of PHA (polyhydroxybutyrate, PHB) from MMC (PHA content of ca. 70%) fed with acetate and compared the results with those obtained from extraction with dichloromethane. These authors verified that the PHA purity in the final product increased when both NaOH concentration and treatment time increased, though PHA recovery decreased in both situations. Applying NaOH at 0.20 M to fresh biomass for 1 h, the authors obtained a PHA purity of 87% and a PHA recovery of 97%. When treatment time was increased to 3 h, PHA purity increased to 92% and PHA recovery was reduced to 94%. The comparison between the results of these alkaline-based methods and the ones obtained with extraction by dichloromethane, 98% of PHA purity and 56% of PHA recovery, suggests that PHA extraction with NaOH might be a viable alternative to extraction with organic solvents.

With the purpose of developing a low-cost extraction method with high PHA recovery to achieve a cost-effective polymer production, Heinrich et al. (2012) [9] studied a simplified method for PHA extraction at large scale from cells of *Ralstonia eutropha* H16 (now reclassified as *Cupriavidus necator*), cultivated with a synthetic carbon/magnesium solution, using sodium hypochlorite (NaClO, 13%, *v/v*). NaClO is a strong oxidizing chemical that dissolves non-PHA cellular mass (NPCM), while PHA granules remain in the solid form [10]. Very high purities have been reported for PHA extraction with NaClO, but as PHA is not completely insoluble in NaClO, the often-associated decrease in polymer molecular weight after extraction has caused concern when dealing with this chemical [9,10,11,12]. In the study by Heinrich et al. (2012) [9], PHA with an average purity of 93% was extracted with a maximum recovery of 87% when the largest extraction volume tested, 50 L, was used. Nevertheless, treatment with NaClO led to a 50–70% decrease in the polymer molecular weight and higher dispersity of the polymer. Villano et al. (2014) [11] recovered PHA (poly(3-hydroxybutyrate-co-3-hydroxyvalerate), PHBV) from fresh MMC, produced using a synthetic mixture of acetic and propionic acids, by operating an extraction reactor using two digestion agents: NaOH (1 M) and NaClO (5% active Cl_2_). In the study by Villano et al. (2014) [11], PHA extraction displayed greater performance when NaClO was used for the digestion of NPCM, as opposed to NaOH, both in terms of polymer recovery (ca. 100%, *w/w*) and purity (>90%, *w/w*). The results showed, however, a wide polymer molecular weight distribution, which might be detrimental to the application of the process. Furthermore, treatments using NaClO may be hazardous and not viable for large-scale application due to the risk of formation of toxic halogenated compounds [5].

Until now, most PHA extraction studies have used either pure cultures or MMC produced with synthetic substrates [1]. The present study focused on the optimization of PHA (PHBV) extraction from a MMC (ca. 70% PHA) produced at pilot scale with fruit pulp waste by NPCM digestion with either NaOH or NaClO. Optimization was performed using lyophilized biomass at a set concentration (20 g/L) through a central composite rotatable design (CCRD), a response surface methodology (RSM) design that efficiently seeks the optimum conditions for a multivariate system. RSM involves performing statistically designed experiments, coefficients estimation in mathematical models and response prediction and model accuracy testing [13]. Central composite design, in particular, has been often used for the optimization of conditions from various processes, including chemical and biochemical reactions [14]. In the current study, CCRD was used to determine different combinations of reagent concentration and digestion time to be tested and also to predict the conditions that maximize both PHA purity and recovery. This study also assessed the impact of intracellular PHA content, biomass pre-treatment and biomass concentration on PHA extraction performance and polymer characteristics, including molecular weight, thermal properties and infrared spectra.

## 2. Materials and Methods

### 2.1. Biomass Samples

Four different biomass samples containing the polymer poly(3-hydroxybutyrate-co-3-hydroxyvalerate) (PHBV) were used in the PHA extraction trials, and all were produced in a three-stage process pilot unit plant operated at room temperature. This unit consisted of an acidogenic fermentation stage, performed in an upflow anaerobic sludge blanket (UASB) reactor with a working volume of 60 L; a culture selection stage, performed in a 100 L sequencing batch reactor (SBR) operating under a feast and famine regime; and a PHA accumulation stage, performed in a 60 L fed-batch reactor. The UASB reactor, the SBR and the fed-batch reactor were designed inhouse. Fruit pulp waste was used as feedstock for this production.

The PHA accumulation process was performed with biomass purged from the culture selection SBR and a volatile fatty acid (VFA)-rich stream derived from the UASB. The accumulation reactor worked in pulse feeding mode under nutrient limitation. After accumulation, the different PHA-enriched biomasses were acidified with sulfuric acid (Sigma-Aldrich, Sigma-Aldrich, Burlington, MA, USA), centrifuged and stored at 4 °C before lyophilization.

Biomass A was collected at the end of a PHA accumulation process and had an intracellular PHA content of ca. 70% (31% of 3-hydroxyvalerate, 3HV). This sample was used for the PHA extraction optimization tests and to study the impact of the biomass concentration on the PHA extraction performance. Biomasses B and C were collected at distinct times of a second PHA accumulation process, namely after the first and second feed pulses, and had PHA contents of ca. 35–41% (18% 3HV) and ca. 44–52% (18% 3HV), respectively. Biomass D was collected at the end of a third PHA accumulation and presented a PHA content of ca. 73% (21% 3HV). Biomasses B, C and D were used to study the effect of the intracellular PHA content on PHA extraction. Additionally, to study the effect of biomass pre-treatment on PHA extraction, trials were performed using biomass C, not only lyophilized but also in its fresh state (i.e., only subjected to acidification and centrifugation) and in its dry state (i.e., subjected to acidification, centrifugation and drying at 60 °C for 3.5 days).

### 2.2. PHA Extraction

For the determination of the optimal conditions for PHA extraction from lyophilized PHA-enriched biomass using NaOH (97%, Sigma-Aldrich, Burlington, MA, USA) or NaClO (10–15% active Cl_2_, Acros Organic, Geel, Belgium), biomass was mixed at 20 g/L in 50 mL tubes with 20 mL of NaOH or NaClO solutions, according to the conditions determined by the experimental design (see Section 2.4), and incubated at 200 rpm and 30 °C. After incubation with NaOH, the suspension was centrifuged at 9300× *g* for 10 min at 20 °C, the resulting pellet was washed with 40 mL of water and then frozen and lyophilized for 48 h. Regarding digestion with NaClO, 20 mL of water were added to the suspension after incubation and before the first centrifugation to enhance the solid–liquid separation. Afterward, the processing of the pellet was the same as previously described for suspensions, including NaOH. For comparison purposes, conventional PHA extraction with chloroform was performed. In this method, 10 g of lyophilized biomass were subjected to Soxhlet extraction with chloroform (99.5% Sigma-Aldrich, Burlington, MA, USA) at 80 °C over 48 h, followed by precipitation in ice-cold ethanol (EtOH; 1:10 *v/v*, Fisher Chemical, Waltham, MA, USA), as described by Pereira et al. (2019) [15].

To assess the effect of biomass pre-treatment on PHA extraction performance, lyophilized, fresh and dry biomasses were mixed at 20 g/L in 50 mL tubes with 20 mL of NaOH or NaClO solutions, using the respective determined optimal conditions, and incubated at 200 rpm and 30 °C. The same experimental conditions were applied to lyophilized biomass with different intracellular PHA contents (biomasses B, C and D) and to lyophilized biomass with the same PHA content (biomass A) but at biomass concentrations of 20, 40, 60, 80 and 100 g/L to analyze the effect of intracellular PHA content and biomass concentration on PHA extraction performance. The processing of the resulting extraction products for both studies followed as described in the first paragraph of the present section.

### 2.3. PHA Content and Composition

PHA content and its composition in biomass and in extraction products were determined by gas chromatography (GC, Trace 1300, Thermo Scientific, Waltham, MA, USA), following a methanolysis method based on the one described by Cruz et al. (2016) [16]. Summarily, the lyophilized samples (1 to 2 mg) were hydrolyzed with 1 mL of 20% (*v/v*) sulfuric acid (Sigma-Aldrich, HPLC grade, Burlington, MA, USA) in methanol (Fisher Chemical, HPLC grade, Waltham, MA USA) and 1 mL of chloroform (Sigma-Aldrich, HPLC grade, Burlington, MA, USA), including heptadecanoate (HD) at 1 g/L, to function as internal standard. The reaction occurred at 100 °C over 3.5 h. The obtained methyl esters were analyzed in a Restek column (Crossbond, Stabilwax, Bellefonte, PA, USA) at a constant pressure of 14.50 Psi, using helium (Air Liquid, Paris, France) as the carrier gas. The oven temperature program was set as follows: 20 °C/min until 100 °C; 3 °C/min until 155 °C; 20 °C/min until 220 °C.

Commercial PHBV (Sigma-Aldrich, 88 mol% 3HB, 12 mol% 3HV, Burlington, MA, USA) was used as standard for the construction of calibration curves that allowed the determination of the mass of both 3HB and 3HV in each sample. The PHA content values were obtained by dividing the mass of PHA (mass of 3HB + mass of 3HV) in each sample by the mass of lyophilized total solids (TS) used for GC analysis, according to Equation (1):

(1) $$\mathrm{PHA}\;\mathrm{Content}\;\left(\%,\frac{\mathrm{mg}}{\mathrm{mg}}\right)=\frac{\mathrm{PHA}}{\mathrm{TS}}\times 100$$

To calculate the PHA recovery yield according to Equation (2), it was necessary to determine the PHA mass of both the final extraction product and of the biomass from which the extraction product was obtained (PHA_final_ and PHA_initial_, respectively).

(2) $$\mathrm{Recovery}\;\left(\%,\frac{\mathrm{mg}}{\mathrm{mg}}\right)=\frac{{\mathrm{PHA}}_{\mathrm{final}}}{{\mathrm{PHA}}_{\mathrm{initial}}}\times 100$$

### 2.4. Experimental Design and Statistical Validation

RSM was used to assess the optimal conditions for PHA extraction using digestion with either NaOH or NaClO. CCRD was performed to analyze the impact and interaction between the experimental variables (X_i_), reagent concentration (M for NaOH and % for NaClO) and digestion time (h), and the observed responses, PHA purity (%) and PHA recovery (%). The design applied consisted of nine experiments performed randomly: four factorial design points at levels ±1; four experiments of axial level α = ±1.414; and a central point with three replicates. The experimental values for the two independent variables were established according to the literature [3,6,8,9,11,12,17,18,19,20,21]. The experimental tests for optimization of PHA extraction with NaOH and NaClO were performed as described in Table 1.

The experimental data were fitted to the second-order model presented in Equation (3) to evaluate the system’s behavior.

(3) $$Y_{p}={\;b}_{0}+b_{1}X_{1}+b_{2}X_{2}+b_{11}X_{1}^{2}+b_{22}X_{2}^{2}+b_{12}X_{1}X_{2}$$

In Equation (3), Y_p_ corresponds to the predicted responses, and X_1_ and X_2_ are the coded values of the independent variables, namely reagent concentration and digestion time. b_0_, b_i_, b_j_, b_ij_ (i, j = 1, 2) are the coefficient estimates, b_0_ being the interception, b_1_ and b_2_ the linear terms, b_11_ and b_22_ the quadratic terms, and the b_12_ the interaction term. A statistical analysis was performed to evaluate the significance of each source of variation and select an appropriate quadratic model.

Analysis of variance (ANOVA) was used to assess the fit of each model, which was considered an accurate prediction tool when it met the following criteria: a good correlation value (R^2^ > 0.7, acceptable for biological samples [22]) with statistical meaning (*p*-value < 0.05, for a 95% confidence level) and with no lack of fit (*p*-value > 0.05, for 95% confidence level) [23]. Statistics and surface plots analysis provided information on the effect of reagent (NaOH or NaClO) concentration and digestion time on PHA purity and PHA recovery.

### 2.5. PHA Infrared Spectra

Fourier transform infrared (FTIR) spectra of the extracted PHA were collected between 400 and 4000 cm^−1^, at room temperature, using a Cary 630 FTIR spectrometer (Agilent Technologies, Santa Clara, CA, USA) with a thermoelectrically cooled dTGS detector and KBr standard beam splitter and equipped with a diamond attenuated total reflectance (ATR) accessory. All spectra were recorded via the ATR method, with a resolution of 1 cm^−1^ and 16 scans.

### 2.6. PHA Molecular Mass Distribution

The weight average molecular weight (Mw), number average molecular weight (Mn) and polydispersity index (Mw/Mn; PDI) of the extracted PHA were determined by size exclusion chromatography (SEC). For this analysis, 15 mg of each sample was first dissolved in 3 mL of chloroform at room temperature for 18 h. Then, the resultant solutions were filtered with glass fiber filters 47 mm (PALL, Port Washington, NY, USA) and analyzed by a Waters SEC system (Milford, MA, USA), with support SEC: PLgel 5 µm Guard, 50 × 7.5 mm; PLgel 5 µm 104 Å, 300 × 7.5 mm; PLgel 5 µm 500 Å, 300 × 7.5 mm (Polymer Laboratories, Church Stretton, UK). A temperature of equilibration of 30 °C was used, along with a flow rate of 1 mL/min, with degassing, and chloroform as the mobile phase. An amount of 100 µL of each sample was injected in the SEC circuit. The refractive index detector Waters 2410 was used for polymer detection, using the sensitivity 512 and a collect duration of 25 min. Relative molecular weights of the polymers were determined according to the universal calibration method adopting polystyrene standards with molecular weights between 800 Da and 504.5 kDa, and using Waters Millenium SEC software (Milford, MA, USA).

### 2.7. PHA Thermal Properties

Differential scanning calorimetry (DSC) analysis was used to probe the thermal properties of the extracted PHA. This analysis was performed using a differential scanning calorimeter DSC Q2000 (TA Instruments, New Castle, DE, USA). Each sample (approximately 4 mg) was placed in a sealed aluminum pan, perforated to allow water/solvents release. Thermograms were collected in a range of temperatures between −90 °C and 160 °C, with heating and cooling steps of 10 °C/min under a nitrogen atmosphere. Two cooling/heating cycles were performed for each sample. The glass transition temperature (T_g_, °C) and the melting temperature (T_m_, °C) were determined as the midpoint of the heat flux step and at the minimum of the endothermic peak, respectively, of the second heating run, due to water or solvent evaporation occurring during the first heating.

## 3. Results

### 3.1. Effects of Reagent Concentration and Digestion Time on PHA Extraction with NaOH/NaClO: Analysis of PHA Purity and Recovery

**Figure 1:**
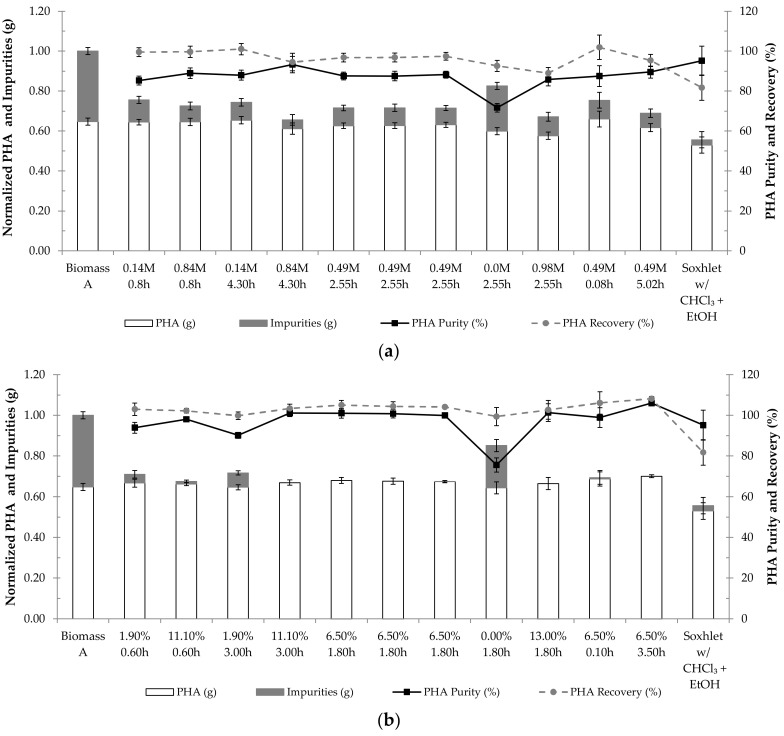
Results of PHA extraction using NaOH (**a**) and NaClO (**b**) for each pair of reagent concentration (**a**) M for NaOH; (**b**) % for NaClO and digestion time (h) tested. The results are expressed in PHA purity (%), PHA recovery (%), normalized mass of PHA (g) and normalized mass of impurities (g). Biomass A corresponds to the starting biomass with intracellular PHA prior to extraction. The results of the conventional PHA extraction with chloroform (extraction with chloroform in soxhlet, followed by purification in ethanol (EtOH)) are also displayed as “Soxhlet w/CHCl_3_ + EtOH”.

With the aim of obtaining the optimal conditions for PHA extraction through NPCM digestion with NaOH or NaClO, a CCRD with reagent concentration and digestion time as independent variables was used to study the PHA purity and PHA recovery of the extraction products. The results from subjecting biomass A (see Section 2.1), lyophilized biomass with ca. 70% of intracellular PHA content, to different digestion conditions with NaOH and NaClO are presented in Figure 1a,b, respectively.

Regarding PHA extraction with NaOH (Figure 1a), the highest PHA purity, 93.28 ± 4.10%, was obtained when the biomass was treated with NaOH at 0.84 M for 4.30 h, and a complete PHA recovery was attained when treatment with NaOH at 0.49 M for 0.08 h was applied. Considering Figure 1b, the final product with the highest PHA purity was obtained, with complete PHA recovery, when the biomass was digested with NaClO at 6.50% for 3.50 h.

ANOVA analysis was used for the two responses (PHA purity and PHA recovery) obtained after digestion with either NaOH or NaClO, and the results are presented in Table 2 and Table 3, respectively.

Table 2 and Table 3 demonstrate R^2^ values greater than 0.7 for PHA purity and PHA recovery obtained after digestion with NaOH and NaClO, respectively. Thus, according to Lundstedt et al. (1998) [22], the second-order model showed an adequate fit for the considered responses. Furthermore, the model and lack of fit *p*-values presented in Table 2 and Table 3 demonstrate that the second-order model had significance (*p* < 0.05) for all responses and no evidence of lack of fit (*p* > 0.05).

### 3.2. Optimal Reagent Concentration and Digestion Time for PHA Extraction with NaOH and NaClO

Multiple linear regression (MLR) analysis of the models developed for PHA extraction using NaOH (Table 4) or NaClO (Table 5) provided information regarding the linear, quadratic and interaction effects of NaOH/NaClO concentration and digestion time on PHA purity and PHA recovery.

Regarding PHA extraction by NaOH digestion, Table 4 shows that PHA purity was affected primarily by the linear and quadratic terms of digestion time (*p* < 0.05). In terms of PHA recovery, this was mainly influenced by the linear terms of NaOH concentration and digestion time, the quadratic terms of digestion time and also the term of interaction between NaOH concentration and digestion time.

Table 5 shows that in PHA extraction through NaClO digestion, PHA purity was mostly impacted by the linear and quadratic terms of NaClO concentration. As for PHA recovery in this process, this was mainly impacted by the linear and quadratic terms of NaClO concentration, as well as the quadratic term of digestion time.

**Figure 2:**
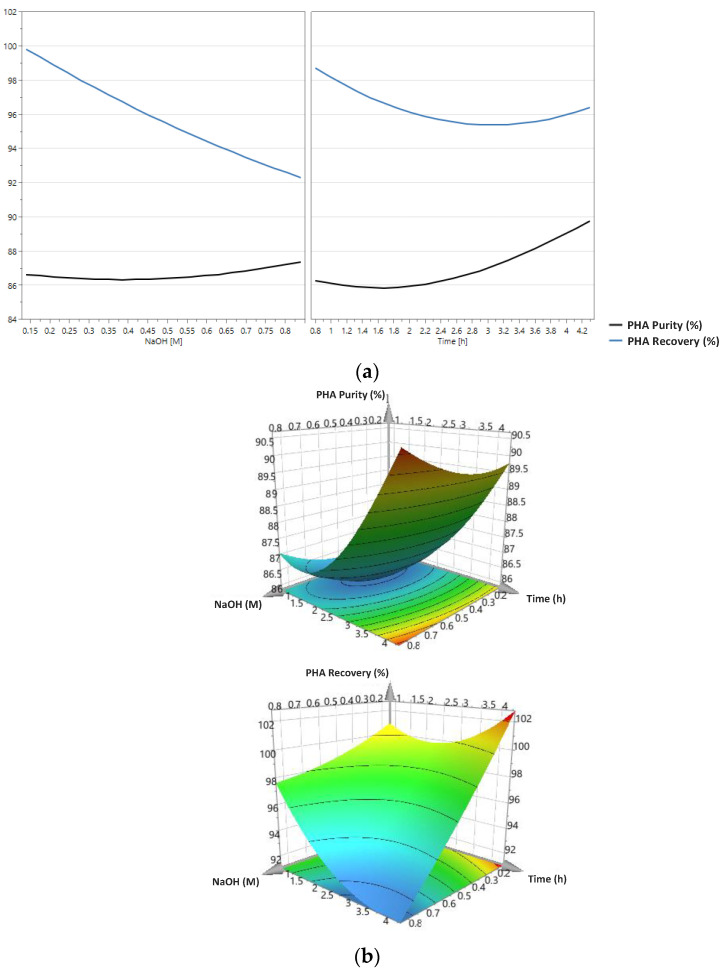
Prediction plots (**a**) and 3D surface plots (**b**) for the models developed for the prediction of PHA purity and PHA recovery considering NaOH concentration and digestion time as independent variables. These models were developed using the experimental PHA purity and PHA recovery values obtained in the CCRD tests for PHA extraction with NaOH.

**Figure 3:**
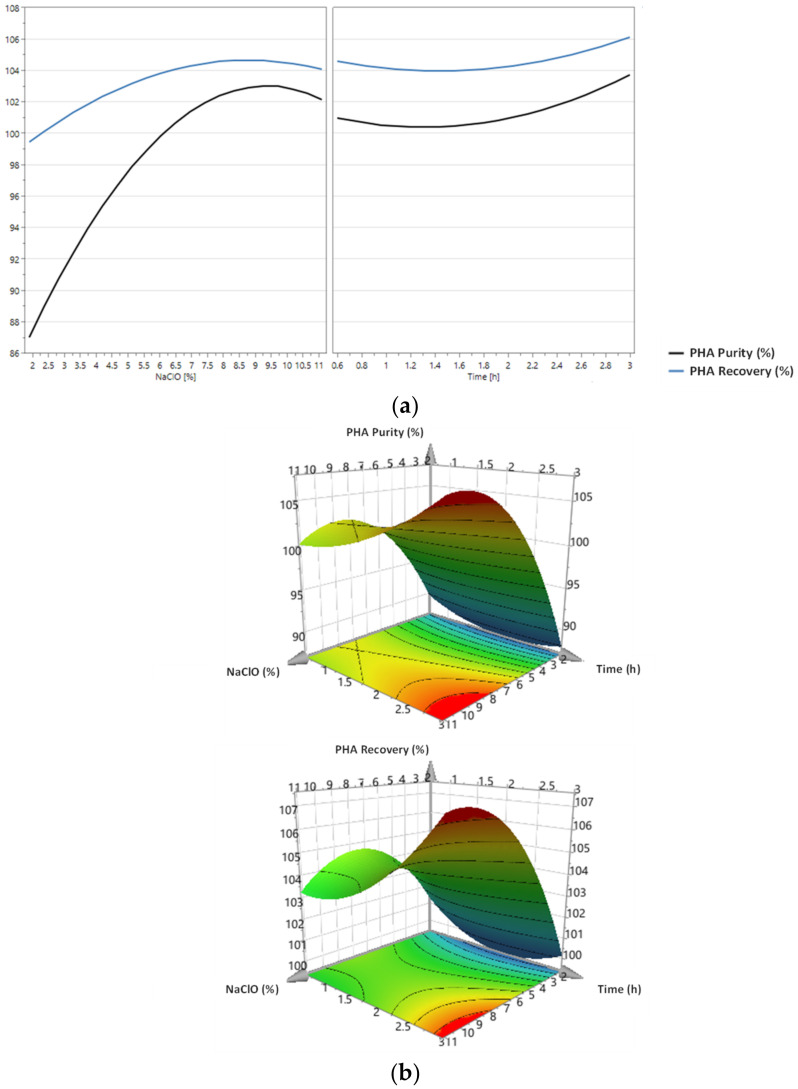
Prediction plots (**a**) and 3D surface plots (**b**) for the models developed for the prediction of PHA purity and PHA recovery considering NaClO concentration and digestion time as independent variables. These models were developed using the experimental PHA purity and PHA recovery values obtained in the CCRD tests for PHA extraction with NaClO.

The prediction plots and the 3D surface plots regarding the PHA purity and PHA recovery models for PHA extraction with NaOH and NaClO are presented in Figure 2 and Figure 3, respectively.

Considering Figure 2a, the developed models suggest that PHA purity slightly increases with the increase in NaOH concentration, while PHA recovery substantially decreases. Concerning the effect of the digestion time, PHA content is expected to increase when the reaction time increases, while PHA recovery will tend to decrease.

Observing Figure 2b, it is possible to assess that PHA content should be maximized when biomass is treated with NaOH at a concentration of about 0.8 M and a digestion time of around 4 h. On the other hand, PHA recovery should be highest when NaOH is used at a concentration of ca. 0.2 M and when the digestion has a duration of about 4 h. 

Considering the developed models, the conditions that should maximize both the PHA purity and the PHA recovery of the final product obtained by PHA extraction with NaOH were estimated at 0.3 M for concentration and 4.8 h for digestion time.

Figure 3a shows that according to the predictive models, PHA content and PHA recovery tend to rise with the increase in NaClO concentration, the increase in PHA purity being greater than the one observed for PHA recovery. In terms of digestion time, both the PHA purity and the PHA recovery are expected to improve as the digestion time increases.

Figure 3b shows that when applying digestion with NaClO, the PHA content of the final product is maximized when NaClO is at a concentration between 8% and 11% and when digestion lasts between approximately 2.7 h and 3 h. Regarding PHA recovery, this parameter should be greatest when the biomass is treated with NaClO at a concentration between 8% and 11% for about 2.8–3 h.

Considering the developed models, the conditions that maximize the PHA content and the PHA recovery of the final product obtained by PHA extraction with NaClO were estimated to be a concentration of 9.0% and a digestion time of 3.4 h.

**Figure 4:**
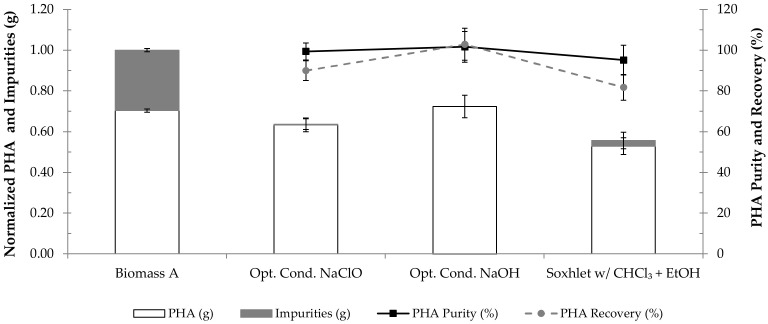
Results of PHA extraction using the optimal conditions for digestion with NaClO (9.0%, 3.4 h) or with NaOH (0.3 M, 4.8 h). The results are expressed in PHA purity (%), PHA recovery (%), normalized mass of PHA (g) and normalized mass of impurities (g). Biomass A corresponds to the starting biomass with intracellular PHA prior to extraction. The results of the conventional PHA extraction with chloroform (extraction with chloroform in soxhlet, followed by purification in ethanol (EtOH)) are also displayed as “Soxhlet w/CHCl_3_ + EtOH”.

Figure 4 shows the results of PHA extraction using the found optimal conditions for NaOH and NaClO digestions, as well as using the benchmark protocol (soxhlet extraction with chloroform followed by precipitation in cold ethanol) for comparison purposes. Digestion with NaClO resulted in a product with a PHA purity of 99.4 ± 4.2%, having recovered 89.9 ± 4.8% of the existing polymer. Treatment with NaOH, under the optimal conditions, recovered 102.9 ± 7.9% of the existing polymer, and the extracted product presented a PHA purity of 101.7 ± 7.6%. On the other hand, soxhlet extraction with chloroform followed by precipitation in ethanol recovered 81.7 ± 6.3% of the polymer and originated a product with 95.1 ± 7.3% of PHA. These results show that treatment with NaClO may have caused some polymer degradation, as it resulted in a lower PHA recovery than digestion with NaOH.

The PHA extraction results presented in Figure 4 suggest that NaOH treatment using the optimal conditions, 0.3 M for 4.8 h, can efficiently extract the polymer from the PHA-enriched biomass used in the present study.

### 3.3. Effect of the Initial Intracellular PHA Content on the PHA Extraction Performance

**Figure 5:**
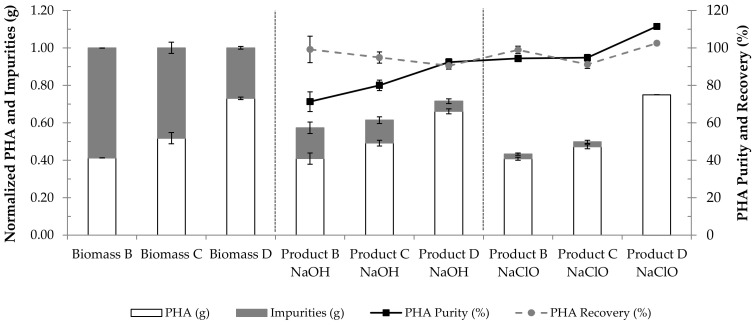
Results of PHA extraction from MMC biomass samples with intracellular PHA contents of 41%, 52% and 73% using the optimal conditions for digestion with either NaOH (0.3 M for 4.8 h) or NaClO (9.0% for 3.4 h). Biomass B, biomass C and biomass D correspond to the starting biomasses with intracellular PHA prior to extraction. Product B, product C and product D correspond to the products extracted from biomasses B, C and D, respectively. The results are expressed in PHA purity (%), PHA recovery (%), normalized mass of PHA (g) and normalized mass of impurities (g).

Studies have shown that the initial PHA content of biomass wields a great influence on the performance of the PHA extraction process [5,24]. It has been estimated that for a PHA extraction process to be cost efficient, the biomass should present a PHA content of over 60%, since when it is under this value, serious complications in the separation process could potentially occur [5,25,26]. To assess the influence of the biomass intracellular PHA content on the performance of the developed PHA extraction methods, three biomass samples produced at pilot scale with the same substrate (fruit pulp waste) but with varying intracellular PHA contents, namely 41%, 52% and 73%, i.e., biomasses B, C and D (see Section 2.1), were subjected to the previously determined optimal conditions for NPCM digestion with NaOH (0.3 M and 4.8 h) and NaClO (9.0% and 3.4 h). The results of these PHA extraction tests are presented in Figure 5.

The results in Figure 5 are in accordance with the literature, as they present a clear influence of the initial intracellular PHA content on the polymer extraction performance. Figure 5 shows that the PHA purity of the extraction products increases with the increase in the initial intracellular PHA content of the biomass, irrespective of the digestion agent applied. Yet, an extraction product without impurities was only obtained when applying the optimal NaClO digestion conditions to the sample with the highest intracellular PHA content tested (73%; biomass D). These results confirm previous findings and allow us to conclude that the more enriched in PHA the biomass is, the purer the extracted polymer will be, translating into greater process efficiency.

### 3.4. Effect of Biomass Pre-Treatment on PHA Extraction Performance

**Figure 6:**
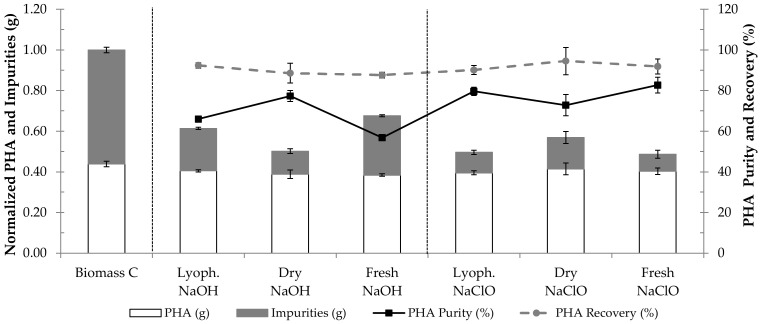
Results of PHA extraction from lyophilized, dry (at 60 °C) and fresh biomass, containing ca. 44% of PHA, using the optimal conditions for digestion with either NaOH (0.3 M for 4.8 h) or NaClO (9.0% for 3.4 h). Biomass C corresponds to the starting biomass with intracellular PHA prior to extraction. The results are expressed in PHA content (%), PHA recovery (%), normalized mass of PHA (g) and normalized mass of impurities (g).

To study the effect of biomass pre-treatment on PHA extraction by NPCM digestion with either NaOH or NaClO, extraction trials were performed with fresh, dried and lyophilized biomass. Biomass C (PHA content of ca. 44%, see Section 2.1) was used in these tests. After having been collected, biomass C was subjected to acidification, centrifugation and storage at 4 °C. This fresh biomass C was subjected to PHA extraction. The dry biomass C was obtained by subjecting the fresh biomass to drying at 60 °C for 3.5 days. The lyophilized biomass C was obtained after lyophilization of the fresh biomass. Figure 6 displays the PHA extraction results of the application of the optimal conditions for either NaOH or NaClO digestion (determined in Section 3.2) to lyophilized, dry and fresh biomass C.

The results of digestion with NaOH in Figure 6 suggest that this method was more efficient when used to process dry biomass rather than lyophilized or fresh biomass, as it resulted in a higher removal of impurities without compromising PHA recovery. While PHA recovery values were similar for all extraction trials with NaOH, ranging between 88 and 92%, the use of dry biomass resulted in a product with a PHA purity of 77%, while NaOH digestion of lyophilized and fresh biomass originated products with PHA purities of 66% and 57%, respectively.

The use of NaClO for PHA extraction from lyophilized, dry and fresh biomass resulted in products with PHA purities of 80%, 73% and 83%, respectively, and similar PHA recovery values, between 90 and 95% (Figure 6). Hence, contrary to PHA extraction by NaOH treatment, digestion with NaClO resulted in products with similar PHA purity and recovery, regardless of the pre-treatment applied to the biomass, though it was slightly less efficient when processing dry biomass.

### 3.5. Effect of Biomass Concentration on PHA Extraction Performance

**Figure 7:**
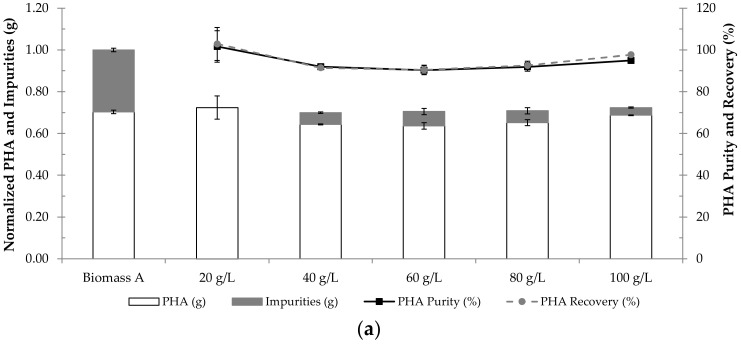

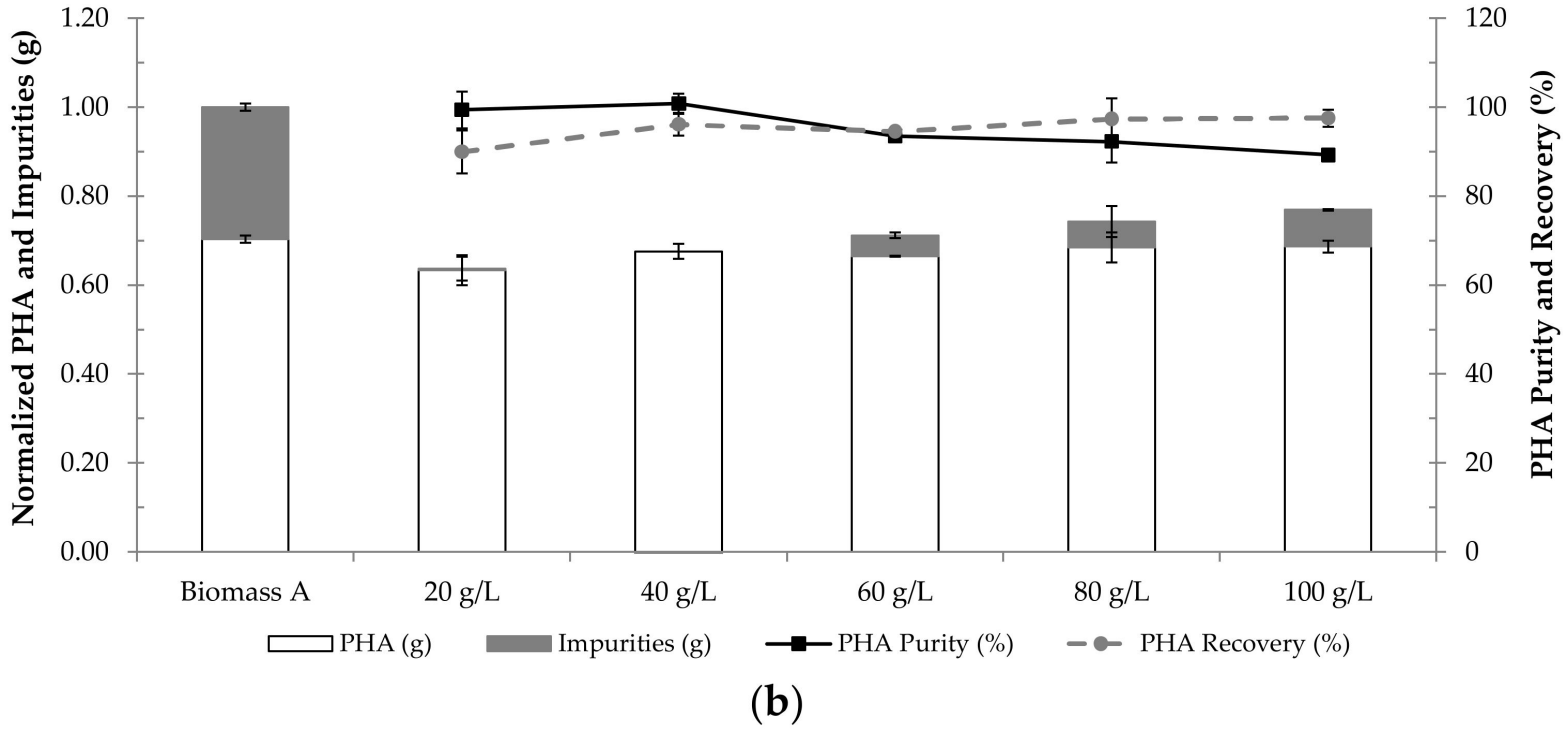
Results of PHA extraction from biomass at the concentrations of 20 g/L, 40 g/L, 60 g/L, 80 g/L and 100 g/L using the optimal conditions for digestion with either (**a**) NaOH (0.3 M for 4.8 h) or (**b**) NaClO (9.0% for 3.4 h). Biomass A corresponds to the starting biomass with intracellular PHA prior to extraction. The results are expressed in PHA purity (%), PHA recovery (%), normalized mass of PHA (g) and normalized mass of impurities (g).

The effect of biomass concentration on PHA extraction by NaOH or NaClO digestion was also studied. For this purpose, biomass A (lyophilized biomass with ca. 70% PHA, see Section 2.1) at the concentrations of 20 g/L, 40 g/L, 60 g/L, 80 g/L and 100 g/L was subjected to the optimal conditions for digestion with NaOH (0.3 M, 4.8 h) or NaClO (9.0%, 3.4 h). The results concerning the effect of biomass concentration on PHA extraction by digestion with either NaOH or NaClO are presented in Figure 7a,b.

Figure 7 shows that at a biomass concentration of 20 g/L, NPCM digestion with either NaOH or NaClO originated products with PHA contents of ca. 100%. When biomass concentration was increased to 40 g/L, the product derived from NaOH digestion presented about 8% of impurities, while the one obtained from NaClO digestion still presented a PHA content of ca. 100%. This suggests that, at their respective optimal conditions, NaClO was able to digest more NPCM than NaOH.

After increasing biomass concentration to 60, 80 and 100 g/L, the PHA purity gradually decreased in the products extracted by NaClO digestion and remained approximately constant in the products extracted by NaOH digestion. Thus, it is possible to conclude that as the biomass concentration increased in the extraction trials using either digestion agent, the PHA purity of the extracted samples tended to decrease, the higher amount of impurities being a consequence of a higher biomass concentration.

### 3.6. Characterization of Extracted Polymers

Selected samples from the PHA extraction tests previously presented were analyzed in terms of Mw, DSC and FTIR spectroscopy. The analyzed samples and the extraction treatments they were obtained from are described in Table 6. The physical-chemical properties of the polymers extracted using the different treatments are presented in Table 7. In terms of physical-chemical properties, sample V will be considered the standard for comparison between samples obtained from the same biomass (lyophilized biomass A with ca. 70% PHA content; samples I, II and V) due to having been obtained using the benchmark protocol for PHA extraction, which, reportedly, causes negligible PHA degradation [27].

#### 3.6.1. Molecular Weight

When comparing the Mw (Table 7) of sample V with that of samples I and II, it is possible to infer that treating lyophilized biomass A with NaOH or NaClO, respectively, resulted in a slight decrease in Mw. This suggests that some polymer degradation occurred during these PHA extraction processes. Nevertheless, samples I and II presented high Mw and values of polydispersity index (PDI) similar to the ones for sample V, around 2, indicating the homogeneity of these polymers.

On the other hand, comparing the Mw and PDI values of samples III and IV suggests that drying biomass C at 60 °C for 3.5 days before extraction resulted in significant changes in the macromolecular features of the polymer, the Mw for sample IV being markedly lower than that for sample III (0.87 × 10^5^ Da and 2.81 × 10^5^, respectively). Furthermore, while sample III presented a PDI of 2.23, sample IV displayed a PDI of 7.12, indicative of a broad molecular weight distribution in the latter.

#### 3.6.2. Thermal Properties

**Figure 8:**
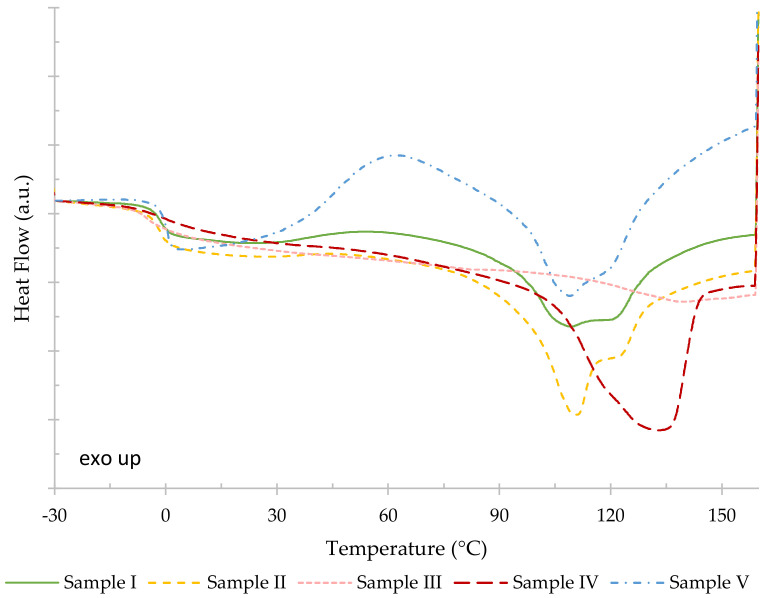
Second heating runs of the DSC thermograms of samples I (green, solid line), II (yellow, dash line), III (pink, short dash line), IV (red, long dash line), V (blue, dash dot line). The thermograms were vertically displaced in order to coincide in the low temperature region.

The thermal properties of each sample, namely glass transition temperature (T_g_), crystallization temperature (T_c_) and respective crystallization enthalpy (ΔH_c_), melting temperature (T_m_) and respective melting enthalpy (ΔH_m_), were determined using the second heating run of the respective thermogram (Figure 8). As moisture/solvent removal occurred during the first heating run, considering the second heating allowed a more accurate comparison between the samples. It should be noted, however, that crystallinity decreased for all samples after the first heating/cooling cycle, as samples recrystallized to a lesser extent. Furthermore, sample III presented a distinctive behavior on the first heating run, being the sample that lost the most mass, attributed to dehydration, the respective water removal endotherm masking the melting.

The second heating run of the thermograms of the considered samples (Figure 8) displays broad melting endotherms, likely due to a wide distribution of crystal thickness and/or size, which influenced their melting temperatures [28]. Furthermore, the thermograms in Figure 8 show that samples I and V undergo cold crystallization above glass transition, revealed by the emergence of a broad exotherm, which could be a consequence of a higher mobility of the polymer chains [29] enabling their ordered arrangement while crossing the glass transition temperature upon heating.

In Table 7, it is possible to observe that the samples with the highest melting temperatures were samples III (138.1 °C) and IV (132.9 °C). This may be due to their lower 3HV content when compared to the other samples, since a melting point decrease in PHBV has been associated with a 3HV content increase [30].

#### 3.6.3. Attenuated Total Reflectance—Fourier Transform Infrared (ATR-FTIR) Spectra

**Figure 9:**
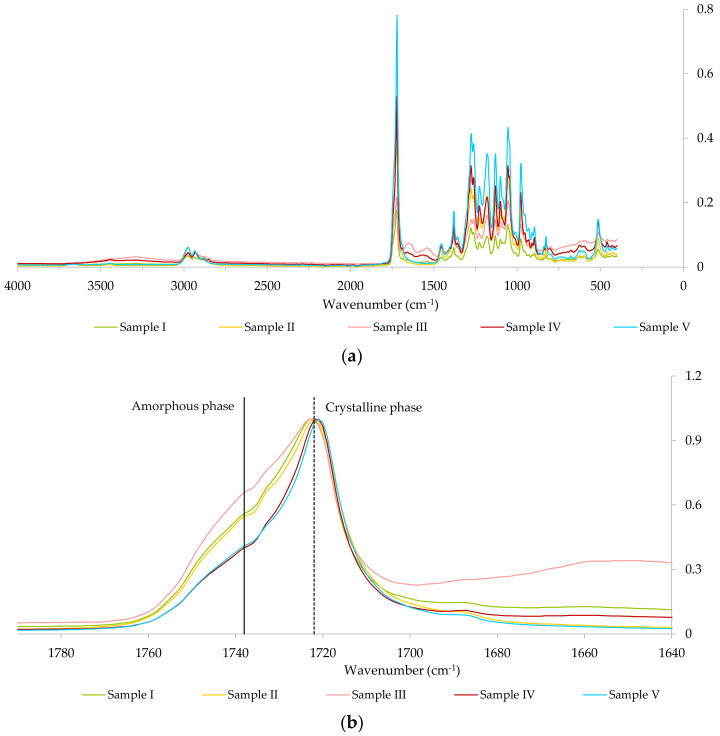
FTIR spectra of the polymer samples obtained using different PHA extraction methods considering: (**a**) the entire wavenumber range (400–4000 cm^−1^); (**b**) a close-up of the carbonyl (C=O) region, 1740–1700 cm^−1^, which includes a broad band at ca. 1738 cm^−1^ and a sharp band at ca. 1722 cm^−1^, assigned to the amorphous and crystalline phases of the polymers, respectively; in (**b**) each FTIR spectra were normalized by the respective maximum absorbance, at ca. 1722 cm^−1^.

ATR-FTIR spectroscopy was used to analyze the selected polymers extracted by NaOH- and NaClO-based methods and also by chloroform extraction followed by EtOH purification (Table 6). The FTIR spectra obtained are presented in Figure 9.

Figure 9a shows that all the samples present the typical PHA bands in FTIR, namely the ester carbonyl band (C=O), stretching in the 1740–1700 cm^−1^ region, and the -CH_3_ and -CH_2_ bands, stretching at 3000–2800 cm^−1^ [31]. In Figure 9b, all FTIR spectra were normalized by their maximum absorbance, at ca. 1722 cm^−1^, to allow a more accurate comparison between the samples in the ester carbonyl band (C=O) region.

Structurally, in the crystalline phase, oxygen atoms of the carbonyl group are located closer to hydrogen atoms, forming hydrogen-bond interactions and leading to a decrease in the carbonyl bond order and to absorbance at lower wavenumbers. On the other hand, the absence of an ordered structure in the amorphous phase leads to reduced hydrogen-bonding effects, resulting in increased carbonyl bond order and absorbance at higher wavenumbers [32], closer to the free C=O stretching mode. Hence, the carbonyl band presents two distinct regions: a relatively broad band at ca. 1738 cm^−1^, which corresponds to the amorphous phase of the polymer, and a sharper band at ca. 1722 cm^−1^, which corresponds to the crystalline phase [33]. Considering this information and Figure 9b, it is possible to infer that sample III exhibits the most amorphous character of all the samples, while the highest crystallinity was found for samples IV and V. The comparison between samples III and IV allows us to conclude that drying the biomass at 60 °C for 3.5 days, prior to the PHA extraction process, influenced the polymer crystallinity in sample IV, since this was the only difference between the pre-treatments of both samples (Table 6). Likewise, as samples I, II and V, which resulted from different extraction methods applied to the same lyophilized biomass, present distinct dynamics between the respective amorphous and crystalline phases, one might assess that the PHA extraction process influences the polymer crystallinity.

## 4. Discussion

In the present study, when the optimal conditions for digestion with NaOH (0.3 M, 4.8 h) were applied to lyophilized biomass (biomass A, PHA content of ca. 70%) produced at pilot scale using fermented fruit pulp as substrate, a PHA purity of 101.7 ± 7.6% and a PHA recovery of 102.9 ± 7.9% were obtained. These values are similar to the ones reported by Jiang et al. (2015) [3], where lyophilized biomass with an intracellular PHA content of ca. 70%, produced with acetate as substrate, was digested using NaOH at 0.2 M for 1 h, resulting in the recovery of 95.5 ± 0.6% of the polymer with a purity of 95.9 ± 3.7%. However, in the current study, a higher NaOH concentration and a longer digestion time were necessary to obtain PHA purity and PHA recovery values similar to the ones reported by Jiang et al. (2015) [3], possibly due to the fact that a real substrate with a more complex matrix was used for biomass production in the case at study. Furthermore, the results of the aforementioned authors suggest that sole NaOH treatment was unable to remove all NPCM from fresh biomass, the remaining impurities requiring the combined action of NaOH and sodium dodecyl sulfate (SDS) to be efficiently removed. In the present study, when testing the effect of different biomass pre-treatments on PHA extraction performance (Figure 6), using biomass with a lower intracellular PHA content (ca. 44%, biomass C), it was also observed that NaOH digestion was less efficient when processing fresh biomass when compared to lyophilized and dry biomass. Hence, one possible approach for future work might be the addition of SDS to NaOH digestion for the extraction of PHA from fresh biomass.

In the present study, the application of the found optimal conditions for NaOH digestion (0.3 M, 4.8 h) to lyophilized biomass with an intracellular PHA content of ca. 41% (biomass B) (Figure 5) resulted in an extraction product with a PHA purity of about 71% and a PHA recovery of ca. 99%. Mohammadi et al. (2012) [8] performed experiments of PHA (PHBHHx) extraction from lyophilized recombinant *C. necator* with a PHA content of ca. 38.3% at 4 °C and 30 °C. At 30 °C, the temperature used in the present study, the highest values of PHA purity and PHA recovery were ca. 95% and ca. 97%, respectively, obtained when biomass was subjected to digestion with NaOH at 0.1 M for 5 h. The lower PHA purity of the final product in the current study may be associated with the fact that MMC are reportedly more resistant to cell hydrolysis than pure cultures [34].

In this study, using the optimal NaOH digestion conditions for the extraction of PHA from fresh biomass C with a PHA content of ca. 44% (Figure 6) resulted in a PHA recovery and a PHA purity of around 88% and 57%, respectively. Villano et al. (2014) [11] performed PHA extraction trials at room temperature using fresh biomass with an average PHA (PHBV) content of 46%, produced with a synthetic mixture of acetic and propionic acids. When subjecting the fresh biomass to NaOH at 1 M (the ratio between the volume of biomass and the chemical solution being 6:1) for 3 h, these authors reported a PHA recovery and a PHA purity of around 87% and 54%, respectively. Using a digestion time of 24 h, a PHA recovery and a PHA purity of about 80% and 56%, respectively, were obtained. These values are similar to the ones obtained in the present study. Regarding digestion with NaClO at 5% Cl_2_ (5.25% NaClO; the ratio between the volume of biomass and the chemical solution being 6:1), the aforementioned authors reported complete recovery of PHA after 3 h and 24 h of digestion. Furthermore, PHA purities of ca. 90% and ca. 98% were obtained after 3 h and 24 h of digestion, respectively. In the present study, subjecting fresh biomass with a PHA content of ca. 44% (biomass C) to the optimal conditions for NaClO digestion (9.0% NaClO, 3.4 h) resulted in a PHA recovery of around 92% and a final product with a PHA purity of ca. 83% (Figure 6). These values are lower than the ones obtained by Villano et al. (2014) [11], which may be due to the fact that the biomass in the current study was produced using real waste as substrate, namely fermented fruit pulp with a complex matrix, while in the former, a synthetic mixture of acetic and propionic acids was used. The use of real waste as substrate may result in the presence of impurities that are harder to remove. On the other hand, the conditions used for PHA extraction from fresh biomass C (PHA content of ca. 44%) with NaClO in the present study were determined using lyophilized biomass with a PHA content of ca. 70%, biomass A (Section 3.2). Thus, it is possible that applying the CCRD methodology to NaClO digestion for PHA extraction from fresh biomass C would determine a different set of optimal conditions that would result in increased PHA purity and PHA recovery. The same possibility can be posed for PHA extraction by NaOH digestion.

Considering Mw results, the polymer obtained from NaClO digestion (9.0%, 3.4 h) of lyophilized biomass with an intracellular PHA content of ca. 70% (sample II in Table 7) presented an average Mw of 2.45 × 10^5^ Da and PDI of 2.20. In the study by Villano et al. (2014) [11], digestion using NaClO, in the conditions mentioned above resulted in a polymer with a Mw range between 3.4 × 10^5^ and 5.4 × 10^5^ Da, and a PDI between 4 and 10. It is possible that the considerable difference between the PDI values of the two studies is due to the process conditions, since in the present study, the polymer was extracted from lyophilized biomass with an intracellular PHA content of ca. 70%, while in the study by Villano et al. (2014) [11], the polymer was extracted from fresh biomass containing ca. 46% of PHA.

Digestion of lyophilized MMC (biomass A, PHA content of ca. 70%) with NaClO, using the optimal conditions (9.0% NaClO, 3.4 h), resulted in a PHA recovery of ca. 90% and a PHA purity of around 99% (Figure 4). Heinrich et al. (2012) [9] extracted PHA (PHB) from lyophilized *R. eutropha* H16 (intracellular PHA content of ca. 65.2%) by subjecting it to digestion with NaClO at 13% (*v/v*) for 1 h at room temperature. In that study, PHA extraction was performed at 0.1 L and 50 L scales. At the scale of 0.1 L, a PHA purity of ca. 95.7% and a PHA recovery of about 91.3% were obtained, whereas at the scale of 50 L, an average of about 87% of the existing polymer was recovered, the final product presenting an average PHA purity of ca. 93%. The reported PHA purity and PHA recovery values using a pure culture are similar to the ones obtained in the current study with an MMC. Nevertheless, in the case at study, PHA extraction by NaClO resulted in a Mw reduction of about 7% when compared to the polymer recovered by chloroform (Table 7), a value notably lower than the one observed by Heinrich et al. (2012) [9], which ranged between 50% and 70%, similarly to previous studies [18,19].

Regarding the effect of biomass concentration on PHA extraction, the results in Figure 7 are in accordance with those obtained by Berger et al. (1989) [19] and Choi and Lee (1999) [6], who observed a decrease in polymer purity when cell concentration was increased in PHA extraction by NaClO and NaOH digestions, respectively. Additionally, Heinrich et al. (2012) [9] also observed that in PHA extraction by digestion with NaClO at 13% (*v/v*), a biomass concentration higher than 30 g/L led to the saturation of the NaClO solution.

Regarding the effect of biomass pre-treatment on PHA extraction, Figure 6 shows that a higher PHA purity was obtained when NaOH digestion was applied to extract PHA from biomass C (intracellular PHA content of ca. 44%) that had been previously dried at 60 °C for 3.5 days, rather than fresh biomass C (ca. 77% of PHA purity in the former vs. ca. 57% in the latter). However, Mw results in Table 7 show that drying biomass C prior to NaOH digestion (sample IV in Table 7) resulted in a major decrease in Mw and increase in PDI when compared to the application of the same process to fresh biomass C (sample III in Table 7). Sample III presented a Mw of 2.81 × 10^5^ Da and a PDI of 2.23, whereas sample IV displayed a Mw of 0.87 × 10^5^ Da and a PDI of 7.12. Lorini et al. (2021) [35] also compared the Mw of polymers extracted from dried and fresh biomass and reported a 3-fold lower Mw for the one extracted from dried biomass. In the present study, it is possible that the drying process resulted in the physical association of the polymer in sample IV with low reversibility upon dissolution. This would justify the higher crystallinity of this sample when compared to sample III, as displayed in Figure 9b, and would explain the decrease in Mw. Considering these results, it is possible to conclude that higher PHA extraction performance does not guarantee superior polymer quality.

DSC and FTIR results presented in Figure 8 and Figure 9b, respectively, suggest that the PHA extraction process influences the polymers’ crystallinity. These results are in agreement with the results obtained by Jiang et al. (2015) [3], which show that chemical treatment and biomass pre-treatment have an impact on the polymer’s crystallinity.

## 5. Conclusions

PHA extraction conditions should be adjusted to the properties of each biomass that is to be processed. In the current study, NaOH and NaClO were tested as green alternatives to organic solvents for the extraction of PHA from MMC biomass produced at pilot scale with fruit pulp waste, and the optimal chemical digestion conditions were determined by a design of experiments methodology. This methodology can be transposed to determine the optimal conditions for PHA recovery from other types of biomasses, subjected to different or no pre-treatments, and with different intracellular PHA contents.

High PHA extraction performances were obtained using the optimal digestion conditions for either NaOH (0.3 M, 4.8 h) or NaClO (9.0%, 3.4 h). However, treatments using NaClO present the risk of formation of toxic halogenated compounds, hindering their application at a large scale. On the other hand, sole digestion with NaOH proved to be an efficient green alternative to chlorinated compounds.

NaClO was similarly efficient for PHA extraction from dry, lyophilized and fresh biomass, though displaying a slightly lower performance for dry biomass, while for NaOH, the greatest efficiency was attained for dry biomass and the lowest for fresh biomass. Digestion of lyophilized biomass with either NaOH or NaClO resulted in negligible loss of polymer molecular weight. However, biomass drying at 60 °C before PHA extraction resulted in a polymer with a broad molecular weight distribution and a decreased mean molecular weight. Furthermore, polymer characterization by DSC and FTIR suggested that its crystallinity is influenced by the applied extraction method.

Intracellular PHA content and biomass concentration were found to strongly influence the PHA extraction performance, irrespective of the digestion agent used. The higher the intracellular PHA content, the higher the PHA purity in the extraction products. On the other hand, when biomass concentration was increased above 20 g/L, the PHA purity of the extraction products tended to decrease. Thus, a compromise between the efficiency of polymer recovery/purity and the process productivity (related to the volume of biomass to be processed) should be considered according to the final polymer application.

## Figures and Tables

**Table 1:** Experimental conditions for the digestion of non-PHA cellular mass (NPCM) with NaOH or NaClO, obtained using a central composite rotatable design (CCRD) of experiments, with reagent concentration (M and % for NaOH and NaClO, respectively) and digestion time (h; t_digestion-NaOH_ and t_digestion-NaClO_ for digestion with NaOH and NaClO, respectively) as independent variables.

Test	NaOH (M)	t_digestion-NaOH_ (h)	NaClO (%)	t_digestion-NaClO_ (h)
1	0.14	0.80	1.90	0.60
2	0.84	0.80	11.10	0.60
3	0.14	4.30	1.90	3.00
4	0.84	4.30	11.10	3.00
5	0.49	2.55	6.50	1.80
6	0.49	2.55	6.50	1.80
7	0.49	2.55	6.50	1.80
8	0.00	2.55	0.00	1.80
9	0.98	2.55	13.00	1.80
10	0.49	0.08	6.50	0.10
11	0.49	5.02	6.50	3.50

**Table 2:** Analysis of variance (ANOVA) of the central composite design applied to PHA extraction through NPCM digestion by NaOH: significance levels (*p*-values) of model and lack of fit, and correlation values (R^2^) for the responses studied, namely PHA purity and PHA recovery.

	Model *p*-Value	Lack of Fit *p*-Value	R^2^
PHA Purity	0.00	0.98	0.82
PHA Recovery	0.00	0.65	0.93

**Table 3:** Analysis of variance (ANOVA) of the central composite design applied to PHA extraction through NPCM digestion by NaClO: significance levels (*p*-values) of model and lack of fit, and correlation values (R^2^) for the responses studied, namely PHA purity and PHA recovery.

	Model *p*-Value	Lack of Fit *p*-Value	R^2^
PHA Purity	0.00	0.06	0.89
PHA Recovery	0.00	0.29	0.80

**Table 4:** Multiple linear regression (MLR) analysis of the polynomial models obtained for PHA extraction through NPCM digestion by NaOH: constants and *p*-values for linear, quadratic and interaction effects of NaOH concentration ([NaOH]) and digestion time (t) for the studied responses, PHA purity and PHA recovery.

Effect	Constant	Linear		Quadratic		Interaction
		[NaOH] (X_1_)	t (X_2_)	[NaOH] × [NaOH] (X_1_^2^)	t × t (X_2_^2^)	[NaOH] × t (X_1_X_2_)
PHA Purity (Y_1_)	−1.134	0.012	0.064	0.020	0.061	0.008
*p*-Value	7.572 × 10^−18^	0.387	8.621 × 10^−5^	0.226	7.859 × 10^−4^	0.619
PHA Recovery (Y_2_)	89.921	−3.535	−1.104	0.479	1.887	−1.985
*p*-Value	6.367 × 10^−24^	2.742 × 10^−7^	0.003	0.284	2.332 × 10^−4^	2.552 × 10^−4^

**Table 5:** Multiple linear regression (MLR) analysis of the polynomial models obtained for PHA extraction through NPCM digestion by NaClO: constants and *p*-values for linear, quadratic and interaction effects of NaClO concentration ([NaClO]) and digestion time (t) for the studied responses, PHA purity and PHA recovery.

Effect	Constant	Linear		Quadratic		Interaction
		[NaClO] (X_1_)	t (X_2_)	[NaClO] × [NaClO] (X_1_^2^)	t × t (X_2_^2^)	[NaClO] × t (X_1_X_2_)
PHA Purity (Y_1_)	100.659	7.572	1.367	−6.079	1.686	2.258
*p*-Value	6.988 × 10^−27^	6.672 × 10^−9^	0.119	1.035 × 10^−6^	0.079	0.100
PHA Recovery (Y_2_)	97.929	2.188	0.728	−2.196	1.202	1.267
*p*-Value	5.869 × 10^−25^	8.157 × 10^−4^	0.160	0.002	0.041	0.088

**Table 6:** Samples selected for analysis of molecular weight (Mw), differential scanning calorimetry (DSC) and Fourier transform infrared (FTIR) spectroscopy and the PHA extraction methods from which these samples were obtained.

Sample	Type of Biomass	PHA Content (%)	Extraction Method
I	lyophilized biomass A	70	NaOH (0.3 M, 4.8 h) + washing with water
II	lyophilized biomass A	70	NaClO (9.0%, 3.4 h) + washing with water
III	fresh biomass C	44	NaOH (0.3 M, 4.8 h) + washing with water
IV	dried biomass C (60 °C, 3.5 days)	44	NaOH (0.3 M, 4.8 h) + washing with water
V	lyophilized biomass A	70	Soxhlet extraction with chloroform + precipitation in absolute EtOH (benchmark protocol)

**Table 7:** Physical-chemical properties of the polymers extracted in selected PHA extraction trials, produced by MMC using fermented fruit waste as feedstock (3HB, 3-hydroxybutyrate; 3HV, 3-hydroxyvalerate; Mw, Mean molecular weight in weight; Mn, Mean molecular weight in number; PDI, Polydispersity index; T_g_, Glass transition temperature; T_c_, Crystallization temperature; ΔH_c_, Crystallization enthalpy; T_m_, Melting temperature; ΔH_m_, Melting enthalpy).

Sample	PHA Purity (%)	3HB in PHA (%wt)	3HV in PHA (%wt)	Mw (×10^5^ Da)	Mn (×10^5^ Da)	PDI	T_g_ (°C)	T_c_ (°C)	ΔH_c_ (J/g)	T_m_ (°C)	ΔH_m_ (J/g)
I	101.7 ± 7.6	69.0	31.0	2.45	1.33	1.84	−1.57	57.1	6.7	109.6	20.8
II	99.4 ± 4.2	69.1	30.9	2.45	1.11	2.20	−1.19	-	-	111.1	27.5
III	56.8 ± 0.8	81.7	18.3	2.81	1.26	2.23	−4.92	-	-	138.1	2.5
IV	77.3 ± 2.7	81.7	18.3	0.87	0.12	7.12	−0.14	-	-	132.9	33.9
V	95.1 ± 7.3	68.8	31.2	2.63	1.31	2.00	0.51	62.1	11.3	109.1	18.5

## Data Availability

Not applicable.
